# Aquaporin 1 and the Na^+^/K^+^/2Cl^−^ cotransporter 1 are present in the leptomeningeal vasculature of the adult rodent central nervous system

**DOI:** 10.1186/s12987-020-0176-z

**Published:** 2020-02-11

**Authors:** Qianliang Li, Nadia N. Aalling, Benjamin Förstera, Ali Ertürk, Maiken Nedergaard, Kjeld Møllgård, Anna L. R. Xavier

**Affiliations:** 1 Center for Translational Neuromedicine, Division of Glial Disease and Therapeutics, University of Copenhagen, 2200 Copenhagen, Denmark; 2 Institute for Stroke and Dementia Research, Klinikum der Universität München, Ludwig Maximilians University of Munich (LMU), 81377 Munich, Germany; 3 Center for Translational Neuromedicine, Division of Glial Disease and Therapeutics, University of Rochester Medical Center, Rochester, NY 14642 USA; 4 Department of Cellular and Molecular Medicine, Faculty of Health and Medical Sciences, University of Copenhagen, 2200 Copenhagen, Denmark

**Keywords:** Aquaporin 1, NKCC1, Subarachnoid space, Leptomeningeal vasculature, Penetrating arterioles, Veins, Capillaries, Venules

## Abstract

**Background:**

The classical view of cerebrospinal fluid (CSF) production posits the choroid plexus as its major source. Although previous studies indicate that part of CSF production occurs in the subarachnoid space (SAS), the mechanisms underlying extra-choroidal CSF production remain elusive. We here investigated the distributions of aquaporin 1 (AQP1) and Na^+^/K^+^/2Cl^−^ cotransporter 1 (NKCC1), key proteins for choroidal CSF production, in the adult rodent brain and spinal cord.

**Methods:**

We have accessed AQP1 distribution in the intact brain using uDISCO tissue clearing technique and by Western blot. AQP1 and NKCC1 cellular localization were accessed by immunohistochemistry in brain and spinal cord obtained from adult rodents. Imaging was performed using light-sheet, confocal and bright field light microscopy.

**Results:**

We determined that AQP1 is widely distributed in the leptomeningeal vasculature of the intact brain and that its glycosylated isoform is the most prominent in different brain regions. Moreover, AQP1 and NKCC1 show specific distributions in the smooth muscle cell layer of penetrating arterioles and veins in the brain and spinal cord, and in the endothelia of capillaries and venules, restricted to the SAS vasculature.

**Conclusions:**

Our results shed light on the molecular framework that may underlie extra-choroidal CSF production and we propose that AQP1 and NKCC1 within the leptomeningeal vasculature, specifically at the capillary level, are poised to play a role in CSF production throughout the central nervous system.

## Background

It is generally accepted that cerebrospinal fluid (CSF) is produced in the ventricular system [1–5] exclusively by the choroid plexus [6–15]. Nonetheless, several studies have challenged the classical view of choroid plexus as the sole source of CSF. After surgical plexectomy of non-human primates, CSF production decreased by only 30%, and the composition of the remaining produced CSF did not differ from that in non-plexectomized rhesus monkeys [16, 17]. Furthermore, the biochemical composition of CSF harvested from the exposed choroid plexus differs from bulk cisternal CSF, suggesting that extra-choroidal sources of CSF exist [4]. Accordingly, studies have suggested that some fraction of CSF production must occur outside the ventricular system in mammals, specifically at the subarachnoid space (SAS) [18–20].

Along with the pia mater, the SAS is a component of the leptomeninges that is filled with CSF, enclosing the brain and spinal cord (reviewed in [21]). In an experimental paradigm in which dogs were perfused with artificial CSF at different pressures into the SAS, quantitative measures indicated that approximately 40% of the total CSF production occurred at this meningeal layer [18–20]. However, that approach gives only a coarse indication of the cellular site and mechanisms underlying extra-choroidal CSF production.

Remarkably, the extensive capillary network present in the CNS has been postulated to contribute to the production of interstitial fluid (ISF), which ultimately mixes with the CSF [4, 22–29]. Fluid filtration from the vascular compartment might occur in the brain capillary endothelia, which contain more mitochondria than the endothelial cells from non-neural tissues [30]. The high content of mitochondria might support active transport of ions at the blood–brain barrier (BBB), which in turn triggers water fluxes, in a manner resembling the mechanism underlying CSF secretion by the choroid plexus. However, the hypothesis that the BBB is a source of CSF presently lacks substantiation from functional studies [29].

Ion pumps, channels and co-transporters in the choroid plexus epithelia drive CSF secretion. Namely, Na^+^ transport occurs via the Na^+^/K^+^ ATPase, and Cl^−^ transport occurs among others via NKCC1, establishing osmotic gradients that result in water movement across the blood-CSF barrier (BCSFB). This passive water movement is especially facilitated by water channels formed by the protein aquaporin 1 (AQP1), which is also expressed by epithelial cells in the choroid plexus (reviewed in [13]). Thus, we asked whether the leptomeningeal vasculature express AQP1 and NKCC1, both proteins involved in CSF secretion by the choroid plexus [11, 13, 31]. Indeed, we observed that both proteins are present in the vasculature distributed within the SAS in the adult rodent brain and spinal cord. The display of the molecular setup involved in CSF production suggest that leptomeningeal vessels may actively contribute to extra-choroidal CSF production throughout the adult rodent CNS.

## Methods

### Animals

We used C57BL/6JRj mice (Janvier Labs, Le Genest-Saint-Isle, France) at postnatal days (P) 30, 60 and 90 (n = 20) of both sexes. Mice were housed in groups (4–5 mice per cage) under a 12-h light–dark cycle and had access to water and standard chow food ad libitum. Additionally, brains were obtained from FVB.Cg-Slc12a2^tm1Ges^/Mmjax, a knockout mice strain for *Slc12a2* gene encoding NKCC1 [32] (P60, n = 2). Paraffin embedded brain sections from 3-month-old Sprague–Dawley rats were also obtained from a previous study [33].

### Antibody characterization (Table 1)

We applied different antibodies against AQP1, recognizing epitopes localized both in the intracellular (rabbit anti-AQP1, Alomone Labs, Jerusalem, Israel and rabbit anti-AQP1 Alpha Diagnostic, San Antonio, TX, USA) and in the extracellular (mouse anti-AQP1, Abcam, Cambridge, UK) domains of the protein. We aimed to improve sensitivity of the immunohistochemistry, minimizing the effects of conformational changes of the target proteins due to fixative cross-linking and treatments such as dehydration and freezing, which might influence epitope availability, especially at the extracellular domain.

**Table 1:** List of primary antibodies

Antigen	Immunogen	Manufacturer (+CAT#)	Host and isotype	Dilution	Reference RRID#
AQP1	(C)KVWTSGQVEEYDLDADDIN Target epitope in mouse AQP1 are amino acids 241 to 261 intracellular	Alomone Labs Cat# AQP-001	Rabbit IgG	1:400	AB_2039726
AQP1	19-aa peptide of Rat AQP1 Target epitope is in C-terminus, intracellular	Alpha Diagnostic Cat# AQP11-A	Rabbit IgG	1:800	AB_1609286
AQP1	(C)SAVLTRNFSN Target epitope in mouse AQP1 are amino acids 199 to 208 extracellular	Abcam Cat# ab11025	Mouse IgG2b	1:500	AB_2289651
NKCC1	Synthetic peptide derived from the N terminal region of human NKCC1 target epitope located intracellularly	Abcam Cat# ab59791	Rabbit IgG	1:500	AB_944433
NKCC1	(C)RRQAMKEMSIDQAK Target epitope in mouse NKCC1 are amino acids 821 to 834 extracellular	Alomone Labs Cat# ANT-071	Rabbit IgG	1:100	AB_2341018
α-SMA	Synthetic peptide corresponding to human alpha smooth muscle Actin (N terminal)	Abcam Cat# ab7817	Mouse IgG2a	1:500	AB_262054
CD31	Tissue, cells or virus corresponding to human CD31	Abcam Cat# ab24590	Mouse IgG	1:500	AB_448167
GAPDH	Source: rabbit muscle (commercially sensitive)	Sigma Aldrich Cat# G8795	Mouse IgM	1:5000	AB_1078991

The rabbit anti-AQP1 (Alomone Labs) detected bands in Western blot analysis corresponding to previously described molecular weights [34–36]. Immunohistochemistry using three different antibodies targeting AQP1 (two rabbit anti-AQP1, from Alomone Labs and Alpha Diagnostic, and one mouse anti-AQP1, from Abcam) showed the characteristic apical expression of AQP1 in choroid plexus, consistent with previous publications [37, 38]. Two antibodies targeting NKCC1, rabbit anti-NKCC1 (Abcam and Alomone Labs) were used and showed expression in choroid plexus consistent with previous studies [31].

The blood vessels were identified with two primary antisera: 1) mouse anti-α-smooth muscle actin (α-SMA; Abcam) and 2) mouse anti-cluster of differentiation 31 (anti-CD31; Abcam), also known as platelet endothelial cell adhesion molecule, PECAM-1. Both antisera stained a pattern of cellular morphology entirely consistent with previous reports for these markers [39, 40].

The band corresponding to the housekeeping protein glyceraldehyde 3-phosphate dehydrogenase (GAPDH) was detected by Western blot using a mouse anti-GAPDH antibody (Sigma-Aldrich, St. Louis, Missouri, USA), with an apparent molecular weight of 37 kDa, as previously described [41, 42].

### Immunohistochemistry

Animals of both sexes (P90; n = 6; 3 males, 3 females) were anesthetized by intraperitoneal (i.p.) injection of a mixture of ketamine and xylazine (100/20 mg/kg) and perfused transcardially with 10 mL 0.01 M phosphate buffer saline (PBS, pH 7.4, Sigma-Aldrich) followed by 30 mL 4% paraformaldehyde solution (PFA, Sigma-Aldrich) diluted in PBS. To label the vascular endothelia, some animals (n = 2) were also perfused with lectin from *Triticum vulgaris* (wheat germ agglutinin, WGA, Sigma-Aldrich, 12.5 μg/mL diluted in PBS, pH 7.4), prior to 4% PFA. Brain, kidney and heart were harvested and post-fixed in 4% PFA overnight. Some mice (n = 4) were decapitated under deep anesthesia without perfusion, and their brains fixed by overnight immersion in 4% PFA at 4 °C. The samples were sectioned using a vibratome (50 or 100 µm thick sections; Leica VT1200S, Wetzlar, Germany). After PBS washes, histological sections were blocked for 1 h at room temperature (RT) in a solution containing 0.3% Triton X-100 (Sigma-Aldrich) and 5% normal donkey or goat serum (Gibco™; Thermo Fisher Scientific, Waltham, Massachusetts, USA) in PBS followed by incubation overnight at 4 °C with primary antibodies (Table 1) diluted in blocking solution. Immunolabeling was revealed by incubation with the appropriate secondary antibodies coupled to fluorophores (Alexa Fluor, 1:500; Invitrogen™ Molecular Probes™; Thermo Fisher Scientific) for 2 h at room temperature. DAPI (4′,6-diamidino-2-phenylindole, Thermo Fisher Scientific, 1 μg/mL diluted in PBS, pH 7.4) was used for nuclear counterstaining prior to mounting with Prolong Gold Antifade Reagent (Invitrogen/Thermo Fisher Scientific, Carlsbad, California, USA). Images of the immunolabeled sections were acquired on a confocal microscope (Nikon Eclipse Ti, Tokyo, Japan) with Plan Fluor 20×/0.75 Mlmm and 40×/1.30 oil objectives or an epifluorescence microscope (Nikon Ni-E) with Plan Apo λ 4×/0.20 objective. Acquired images were adjusted for brightness and contrast using FIJI/ImageJ software.

In addition, we used paraffin sections obtained from mice and rats, processed according to standard protocols. Endogenous peroxidase activity was first quenched by immersion in a 0.5% solution of hydrogen peroxide in TRIS buffered saline (TBS, 5 mM Tris–HCl, 146 mM NaCl, pH 7.6) for 15 min. After rinsing with TBS, non-specific binding was inhibited by incubation for 30 min with 10% goat serum (Biological Industries, Kibbutz Beit-Haemek, Israel) at room temperature. Next, sections were incubated overnight at 4 °C with the anti-AQP1 primary antibodies (Alpha Diagnostic; 1:800 and Alomone Labs; 1:400) diluted in 10% goat serum and washed with TBS. For bright field light microscopy analysis, the REAL™ EnVision™ Detection System, Peroxidase/Diaminobenzidine + (DAB+) rabbit/mouse (K5007, Dako, Glostrup, Denmark) was used for detecting the primary antibodies. The detection reagent consists of a dextran backbone coupled to peroxidase and polyclonal secondary antibody molecules. The sections were washed with TBS, followed by incubation for 10 min with the DAB + solution. Sections were counterstained with Mayer`s hematoxylin, dehydrated in graded alcohols and cover-slipped with Pertex mounting medium. Additionally, Bouin’s fixed paraffin embedded coronal sections (5 µm thick) of 3-month-old Sprague–Dawley rat brains were selected from serially sectioned tissue obtained from a previous study [33].

### Tissue clearing

Mice of both sexes (P60; n = 4) were perfusion-fixed with 4% PFA, and the brains harvested as described above for immunohistochemistry. We used young adult mice, which have a less rigid skull, allowing a more efficient penetration of the antibodies into the brain parenchyma. The whole mice heads were immunolabeled and cleared using the uDISCO protocol [43]. In brief, fixed tissue was serially dehydrated in methanol (Sigma-Aldrich), and then bleached by immersion in ice-cold 5% H_2_O_2_ (Sigma-Aldrich) containing 20% dimethyl sulfoxide (DMSO, Sigma-Aldrich) in methanol at 4 °C overnight (o/n). After bleaching, samples were rehydrated for immunolabeling, with all steps carried out at 37 °C. First, whole heads were permeabilized by o/n incubation in 20% DMSO, 0.3 M glycine (Sigma-Aldrich) and 0.2% Triton X-100 in PBS. Samples were blocked in 20% DMSO, 6% normal donkey serum and 0.2% Triton X-100 in PBS for 1 day. Prior to primary antibody incubation, samples were washed with PBS added with Tween-20 and heparin (PTwH; 0.2% Tween-20, Sigma-Aldrich with 10 mg/mL heparin, Thermo Fisher Scientific diluted in PBS) o/n. Primary antibody (anti-AQP1int, Alomone; 1:400) was diluted in PTwH containing 5% DMSO and 3% normal donkey serum. Incubation was performed for 14 days and the primary antibody solution was refreshed every third or fourth day. After the primary antibody incubation period, samples were washed thoroughly with PTwH and incubated with appropriate secondary antibody diluted in 3% normal goat serum in PTwH for 8 days, refreshing every second day. Final washes were done with PTwH. For tissue clearing, a mixture of benzyl-alcohol and benzyl benzoate (BABB, dilution ratio 1:2; Sigma Aldrich) was applied, and the samples dehydrated by serial incubations with tert-butanol (Sigma Aldrich), diluted in distilled water when applicable. After dehydration, the samples were delipidated by incubation in dichloromethane (DCM; Sigma Aldrich) for 1 h followed by a 2 h incubation in BABB mixed with diphenyl ether (Alfa Aesar) 4:1 (BABB-D4) along with 0.4% d,l-α-tocopherol (Vitamin E; Thermo Fisher Scientific). Samples were then stored in BABB-D4 at room temperature in the dark until imaging with a light-sheet microscope (LaVision BioTec UltraMicroscope II, Göttingen, Germany), using an Olympus 2X/0.15 NA (WD 10 mm) objective. Image analysis was performed using Imaris software (BitPlane, Belfast, UK).

### Western blot

Animals of both sexes (P30; n = 6; 3 males and 3 females) were anesthetized by i.p. injection of a mixture of ketamine and xylazine (100/20 mg/kg) and perfused transcardially with 10 mL of Hank’s Balanced Salt Solution (HBSS, Gibco™; Thermo Fisher Scientific). Young mice (P30) were used in order to minimize the time of tissue harvesting, thus preventing protein degradation of the different regions analyzed in the present study. Brains were removed from the skull and the different regions analyzed in this study—brain stem, cerebellum, choroid plexus, cortex, hippocampus, hypothalamus and olfactory bulb—were quickly dissected under a stereomicroscope (SMZ1270; Nikon) and snap frozen in dry ice. The right kidney of each mouse was harvested and used as a positive control for the assessment of AQP1 protein levels. Samples were stored at − 80 °C until homogenization with 12.5% protease inhibitor solution (cOmplete, Mini, EDTA-free Protease Inhibitor cocktail; Roche, Basel, Switzerland) in lysis buffer (50 mM Tris–HCl, 1% NP-40, 0.5% sodium deoxycholate, 5 mM EDTA, 150 mM NaCl diluted in water; Thermo Fisher Scientific). Total protein concentrations were determined using BCA assay (Kit BCA™ Protein Assay Kit; Sigma-Aldrich). Proteins from the different tissue homogenates (80 µg/mL of total protein content) were resolved under reduced conditions (NuPAGE™ Sample Reducing Agent; Thermo Fisher Scientific) by SDS-PAGE (Invitrogen™ Novex™ NuPAGE™ 4 - 12% Bis–Tris Protein Gels; Thermo Fisher Scientific) using MOPS SDS running buffer (NuPAGE™, Thermo Fisher Scientific). Proteins were subsequently transferred onto nitrocellulose membranes (Amersham Protran Premium 0.45 µm NC; GE Healthcare Life Sciences, Chicago, Illinois, USA). For AQP1 and GAPDH detection, membranes were blocked using Pierce Clear Milk Blocking Buffer (Thermo Fisher Scientific) diluted in wash buffer (0.25 M Tris Base, 1.7 M NaCl, 0.5% Tween-20 diluted in water, pH adjusted to 7.6) for 2 h at room temperature under agitation, and then incubated with appropriately diluted primary antibodies at 4 °C o/n (see Table 1). The membranes were washed three times for 10 min with washing buffer before incubation with the secondary antibodies diluted in blocking buffer for 2 h at room temperature. After washing, the membranes were imaged with ChemiDoc™ MP (Bio-Rad, Hercules, California, USA) using 530/28 nm and 695/55 nm emission filter/bandpass. The intensity of the detected bands was determined using ImageJ gel analyzer by subtracting the background. Band intensities were normalized to those for GAPDH.

### Statistical analysis

The AQP1/GAPDH ratios obtained from three independent Western blot experiments were analyzed using R 3.4.0 [44]. Each band, corresponding to the AQP1 protein content of different brain regions, was normalized by dividing by the sum of all bands on the same blot, as previously described [45]. Bar plot results are expressed as mean $\pm$ SEM, and differences between multiple regions were assessed via Kruskal–Wallis test. A *p* value < 0.05 was considered significant for rejection of the null hypothesis.

## Results

### Aquaporin 1 is widely expressed in the adult rodent brain in its glycosylated and non-glycosylated forms

**Fig. 1:**
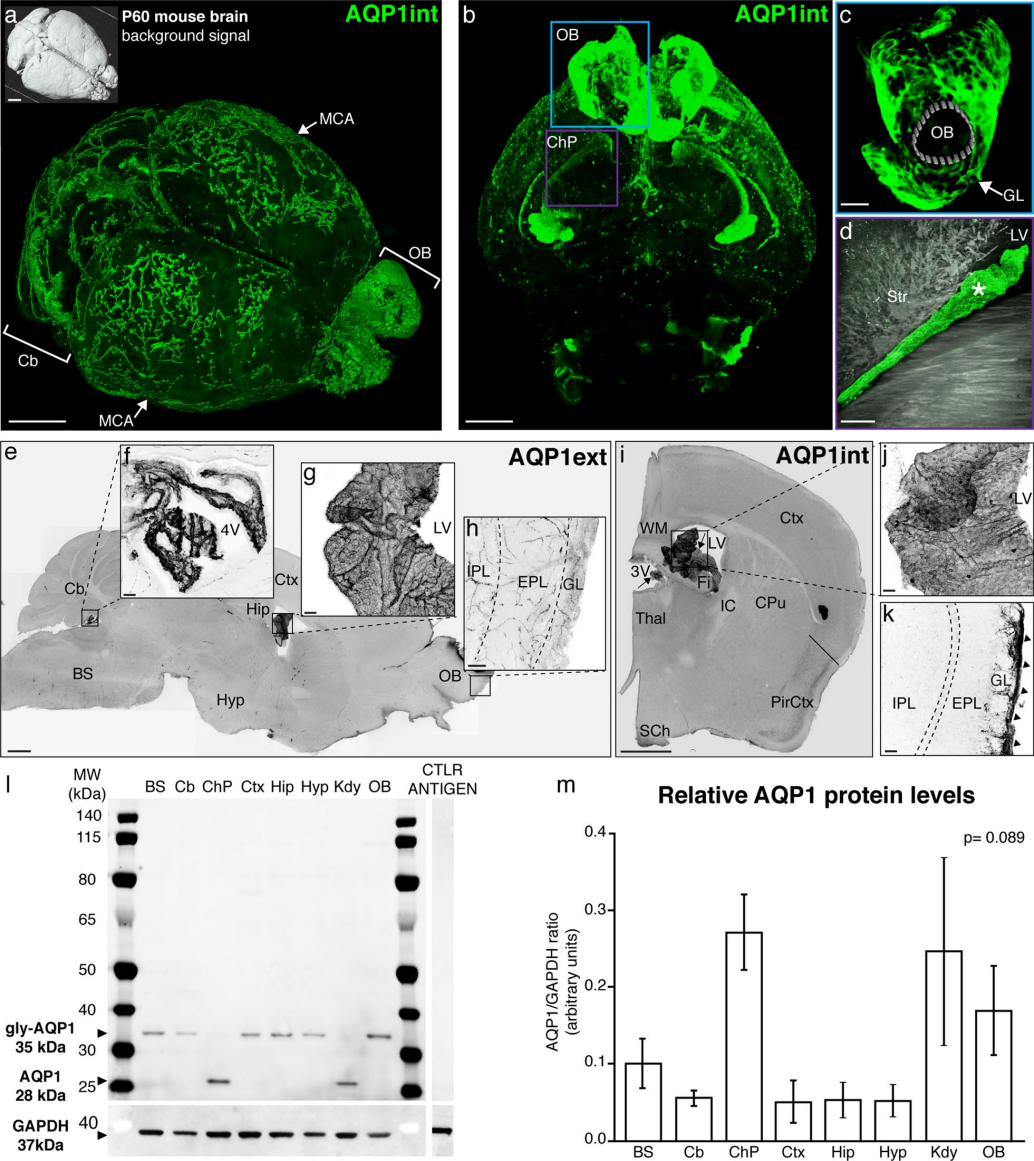
uDISCO clearance of the intact mouse head depicts the expression of aquaporin 1. **a** Mouse brain (P60) cleared by uDISCO and immunolabeled for AQP1 (AQP1int, green) reveals the vasculature network in the leptomeninges, including the middle cerebral arteries (MCA, arrows). AQP1^+^ cells also line the subarachnoid cisterns and the olfactory bulb. **b** Optical section reveals AQP1^+^ choroidal epithelial cells and olfactory ensheathing glia cells. **c**, **d** Higher magnification images of the areas depicted in **b** (blue and purple squares) showing AQP1 in the glomerular layer (arrow) and in choroidal epithelial cells (asterisk). **e** Representative micrograph of a parasagittal section of an adult mouse brain (P90) immunolabeled for AQP1 (AQP1ext, grey). AQP1ext^+^ epithelial cells of the choroid plexus are observed in the fourth (**f**) and in the lateral ventricles (**g**). In contrast, olfactory ensheathing glia cells in the olfactory bulb are not immunolabeled (**h**). **i** Representative micrograph of a coronal section from adult mouse brain (P90) immunolabeled with AQP1 (AQP1int, grey). **j** Higher magnification of the depicted area in **i** (square) shows in detail AQP1int^+^ epithelial cells in the choroid plexus of the lateral ventricles. **k** Olfactory ensheathing glia cells are also immunoreactive. Dashed line in **k** depicts the mitral cell layer. **l** Immunoblotting reveals a band of 35 kDa, corresponding to the glycosylated form of AQP1, detected in the BS, Cb, Ctx, Hip, Hyp and OB, obtained from young adult mice (P30). The non-glycosylated form of AQP1, corresponding to a band of 28 kDa, is detected in choroid plexi and kidney homogenates obtained from young adult mice (P30). The housekeeping protein GAPDH (37 kDa) was used as loading control. Control antigen confirms antibody-epitope specific binding. **m** Graphic shows the relative AQP1 protein levels, in relation to GAPDH. BS, brain stem; Cb, cerebellum; ChP, choroid plexus; Ctx, cerebral cortex; CPu, caudate putamen; EPL, external plexiform layer; Fi, fimbria; GAPDH, glyceraldehyde 3-phosphate dehydrogenase; GL, glomerular layer; Hip, hippocampus; Hyp, hypothalamus; IC, internal capsule; IPL, internal plexiform layer; Kdy, kidney; LV, lateral ventricle; OB, olfactory bulb; PirCtx, piriform cortex, SCh, suprachiasmatic nuclei; Thal, thalamus; WM, white matter; 3V, third ventricle; 4V, fourth ventricle. Scale bars: **a**, **b**, **e** 1 mm; **c**, **i** 500 μm; **d**, 200 μm; **f**–**h**, **j**, **k** 50 μm

Using light-sheet microscopy we assessed the distribution of AQP1 in the intact adult mouse brain (P60; n = 4) upon uDISCO tissue clearing technique. The use of an antibody that recognizes an epitope in the intracellular domain of AQP1 (AQP1int) revealed immunoreactive cells in the subarachnoid cisterns adjacent to the cerebellum, in the leptomeningeal vasculature, notably along the middle cerebral arteries (MCAs), and in the olfactory bulb (Fig. 1a). As previously described [46], AQP1^+^ cells were restricted to the outer layer of the olfactory bulbs (Fig. 1b, c), corresponding to olfactory ensheathing glia cells that surround the glomeruli. Also in accordance to previous studies [47–49], AQP1^+^ epithelial cells were observed in the choroid plexus (Fig. 1b, d).

In order to confirm and extend these findings, we next employed two distinct antibodies to compare the regional profiles by which they label AQP1 in the adult mouse brain (P90; n = 6). In addition to AQP1int, we used an antibody that recognizes an epitope in the extracellular domain (AQP1ext). Our analysis showed that AQP1ext^+^ epithelial cells are present in the choroid plexus epithelium located in the fourth and the lateral ventricles (Fig. 1e–g). No immunoreactivity was detected in the glomerular layer of the olfactory bulb using the AQP1ext antibody (Fig. 1h). In contrast, anti-AQP1int immunolabeled the epithelial cells of the choroid plexus located in the third and lateral ventricles (Fig. 1i, j), and the ensheathing glial cells surrounding the glomerular layer of the olfactory bulb (Fig. 1k).

We next accessed AQP1 protein content in different brain regions by Western blotting. Using the anti-AQP1int antibody, we quantified the AQP1 protein levels in tissue homogenates obtained from the brain stem (BS), cerebellum (Cb), choroid plexus (ChP), cerebral cortex (Ctx), hippocampus (Hip), hypothalamus (Hyp) and olfactory bulb (OB). We used kidney homogenate (Kdy; P30; n = 2 animals, 1 male and 1 female) as a positive control [50]. The immunoblotting analysis detected two bands, corresponding to the non-glycosylated (28 kDa) and glycosylated (35 kDa) forms of AQP1 [34, 51] (Fig. 1l). Under reducing conditions, homogenates obtained from the different brain regions showed different levels of the glycosylated form of AQP1 (Fig. 1l, m). In sharp contrast, the non-glycosylated form of AQP1 was the prevalent form in homogenates obtained from the choroid plexus and kidney. AQP1 was detected in all brain regions analysed, but no significant regional differences were observed (BS: 0.10 ± 0.03; Cb 0.06 ± 0.01; ChP: 0.27 ± 0.05; Ctx: 0.05 ± 0.03; Hip: 0.05 ± 0.02; Hyp: 0.05 ± 0.02; Kdy: 0.24 ± 0.12; OB: 0.17 ± 0.06; mean ± SEM; Kruskal–Wallis test, H = 12.36, df = 7, p = 0.089; n = 3 independent experiments).

### AQP1 is expressed in the vasculature of the adult rodent brain

**Fig. 2:**
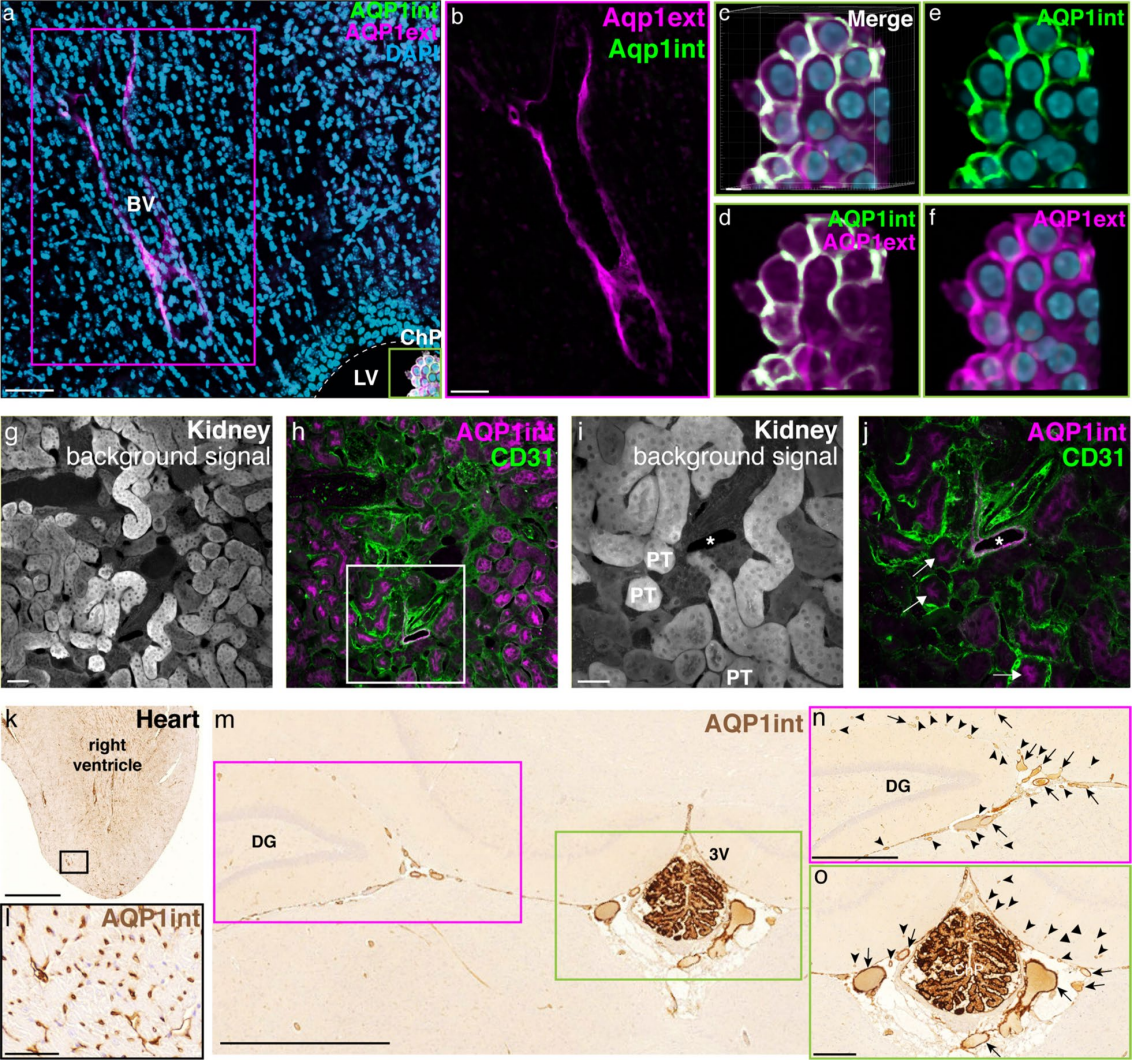
AQP1 is expressed in the brain and peripheral vasculature. **a** Confocal micrograph from an adult mouse brain (P90) immunolabeled for AQP1 (AQP1ext, magenta and AQP1int, green). DAPI nuclear counterstaining (blue). **b** AQP1ext^+^ blood vessel, located around the ventricles (delimited by the magenta square in **a**). **c**–**f** Immunoreactive epithelial choroid plexus cells, located in the lateral ventricles, are labeled with both antibodies (high magnification of the area delimited by the green square in **a**). **g**, **h** Micrographs of mouse kidney show the distribution of AQP1 in the vascular endothelium and proximal tubules. **i**, **j** Higher magnification image of a blood vessel immunolabeled for CD31 (green) and AQP1int (magenta) (delimited by square in **h**). Asterisk indicates the lumen of a blood vessel and arrows indicate proximal tubules. **k**, **l** AQP1^+^ endothelial cells are also detected in the heart of adult mice. **m**–**o** Paraffin sections obtained from adult rat brain show AQP1 immunoreactive blood vessels in the hippocampal fissure and epithelial cells of the choroid plexus located in the third ventricle. Arrows and curved arrowheads indicate arterioles or veins and capillaries or venules, respectively. Straight arrowheads indicate AQP1^−^ blood vessels. 3V, third ventricle; BV, blood vessel; ChP, choroid plexus; DG, dentate gyrus; LV, lateral ventricle; PT, proximal tubule. Scale bars: **a**, **b** and **g**–**j** 50 µm; **c**–**f** 5 µm; **k** 1 mm; **l** 100 µm; **m** 2 mm; **n** 500 μm; **o** 200 μm

In contrast to what is observed in the peripheral vasculature, studies have reported that endothelial cells in the CNS are devoid of AQP1 expression [52–54]. In view of our present results showing marked AQP1 distribution in the leptomeningeal vasculature, and given that we find that the glycosylated form predominates in brain regions other than the choroid plexus and olfactory bulbs, we sought to confirm the presence of AQP1^+^ cells in the CNS vasculature. Adult mouse brain sections (P60; n = 6 animals) were double-labelled for anti-AQP1int and anti-AQP1ext. Confocal microscopy imaging revealed AQP1ext^+^ blood vessel-like structures located next to the ventricular system (Fig. 2a, b), close to the AQP1ext^+^/AQP1int^+^ choroid plexus epithelial cells (Fig. 2a, c–f). Histological sections obtained from adult mouse kidney were immunolabeled for CD31 and AQP1. As previously described [55, 56], epithelial cells in the proximal tubules are AQP1^+^, as well as renal vascular endothelial cells (Fig. 2g–j). Endothelial cells distributed in the adult mouse heart vasculature were also immunoreactive for AQP1int (Fig. 2k, l). AQP1^+^ blood vessels were also observed in the adult rat brain, lining the hippocampus, along with AQP1^+^ epithelial cells and blood vessels of the choroid plexus located in the third ventricle (Fig. 2m–o). Hence, we find AQP1 expression in cerebral blood vessels, notably those in close proximity to the ventricular system and lining the hippocampus, in addition to expression in the leptomeningeal vasculature in the SAS observed along the surface of the adult mouse brain.

### AQP1 and NKCC1 are both expressed by the choroid plexus epithelial cells and the leptomeningeal vasculature

The Na^+^/K^+^/2Cl^−^ cotransporter 1 (NKCC1) is present in the choroidal epithelial cells and has been implicated in CSF production due to its capacity to couple water movement to ion translocation [13, 31, 57, 58]. Previously described in vitro [59], the presence of NKCC1 on endothelial cells has been confirmed in the adult rat brain tissue [60, 61].

**Fig. 3:**
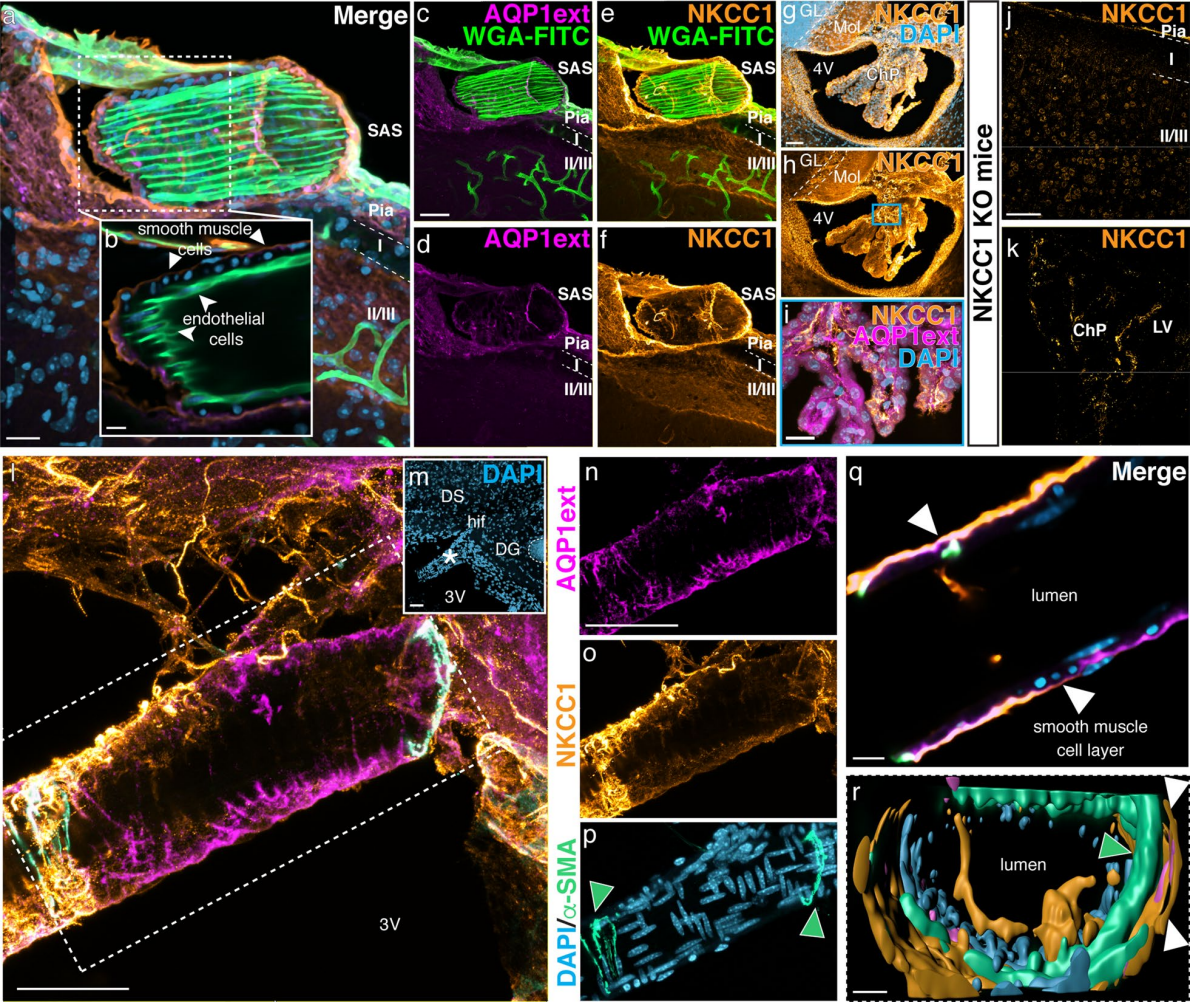
AQP1 and NKCC1 are expressed by the choroidal epithelial cells and in the leptomeningeal vasculature. **a**–**f** Confocal micrograph show a leptomeningeal WGA-FITC^+^ (green) labeled vessel immunoreactive for AQP1 (magenta) and NKCC1 (orange) in the adult mouse brain (P90). In **b** an optical section reveals that AQP1^+^/NKCC1^+^ cells are restricted to the smooth muscle cell layer (arrowheads) and absent in the endothelial cells (curved arrowheads), which are labeled by WGA-FITC. **g**, **h** NKCC1 is detected in the choroid plexus epithelia, in ependymal cells and in the molecular layer of the cerebellum, as shown in the micrographs of the fourth ventricle. **i** Double labeling confirms AQP1 and NKCC1 presence in choroid plexus epithelial cells (higher magnification of the area delimited by the blue square in **h**). **j**, **k** Brain sections obtained from NKCC1 KO adult mice show no immunoreactivity in the brain parenchyma neither in the choroid plexus. **l**, **m** Histological sections immunolabeled with antibodies against AQP1ext (magenta), NKCC1 (yellow) and α-SMA (cyan), reveal AQP1ext^+^/NKCC1^+^/α-SMA^+^ leptomeningeal vessels around the hippocampus and third ventricle. Low magnification micrograph shows DAPI (blue) counterstaining and indicates a leptomeningeal blood vessel (asterisk) closely located to the hippocampal fissure. **n**–**p** Higher magnification of an AQP1ext^+^/NKCC1^+^ vessel (delimited by the dashed square in **j**. Arrowheads indicate α-SMA^+^ cells. (**q**) Optical sectioning reveals that both AQP1 and NKCC1 are distributed in the smooth muscle cell layer (arrowheads). **r** 3D rendering of the leptomeningeal vessel confirms AQP1 and NKCC1 restriction to the smooth muscle cell layer (arrowheads). ChP, choroid plexus; DG, dentate gyrus; DS, dorsal subiculum; GL, granular layer; hif, hippocampal fissure; Mol, molecular layer; SAS, subarachnoid space; 3V, third ventricle, 4V, fourth ventricle. Scale bars: **a**, **i** 20 µm; **b**–**f**, **q**, **r** 10 µm; **g**, **h**, **j**–**p** 50 µm

Confocal microscopy analysis of histological sections obtained from adult mice brain showed that leptomeningeal blood vessels are immunoreactive for both AQP1 and NKCC1 (Fig. 3a, c–f). Optical sections further revealed that NKCC1 as well as AQP1 are distributed along the smooth muscle cell layer, yet not expressed by endothelial cells of arterioles and/or veins (Fig. 3b). NKCC1 was additionally detected in ependymal cells lining the ventricular walls, and in cerebellar neurons located in the molecular and Purkinje layers (Fig. 3g, h), and as previously described [31, 62], NKCC1 was detected in choroidal epithelial cells, which are also AQP1^+^ (Fig. 3i). Brain sections obtained from NKCC1 KO mice showed no immunoreactivity for NKCC1 (Fig. 3j, k). Blood vessels in the hippocampal fissure are also immunoreactive for AQP1 and NKCC1 (Fig. 3l, m). Leptomeningeal vessels observed in this region presented AQP1 and NKCC1 immunoreactivity along their smooth muscle cell layer (Fig. 3n–r).

### AQP1 and NKCC1 are present in the arteriolar and venous smooth muscle cell layer and in the endothelia of capillaries and venules of the leptomeningeal vasculature in the CNS

**Fig. 4:**
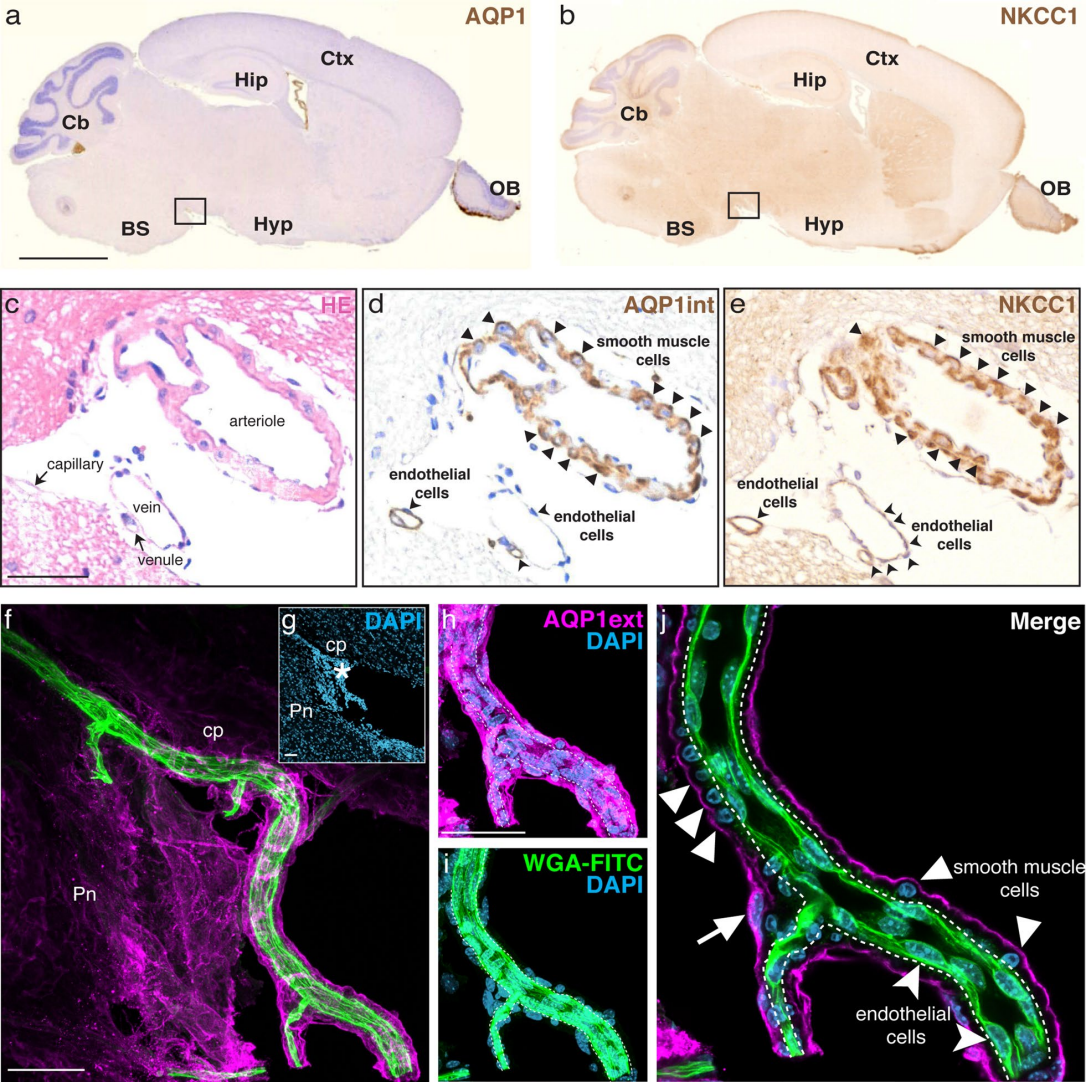
AQP1 and NKCC1 are present in smooth muscle and endothelial cells of the leptomeningeal vasculature. **a**, **b** Paraffin sections of adult mouse brain (P90) immunolabeled with anti-AQP1int or anti-NKCC1 (both brown). **c** Some sections were stained with hematoxylin (HE, pink) and the vascular identity of blood vessels located in the subarachnoid space (cisterna interpendicularis, delimited by square in **a**, **b**) was determined. **d**, **e** Consecutive sections show that AQP1int^+^/NKCC1^+^ cells are present in the smooth muscle cell layer of arterioles (arrowheads) and in the endothelium of capillaries and venules, respectively (curved arrowheads). **f**, **g** Vascular endothelial cells were labeled by lectin (WGA-FITC, green), followed by standard Immunolabeling. DAPI counterstain (blue) reveal the location of the leptomeningeal vessel (asterisk). **h**–**j** Higher magnification confocal images show that AQP1 is restricted to tunica media, where AQP1ext^+^ smooth muscle cells, identified by their round soma (arrowheads) are observed, whereas AQP1 is not present in the endothelial cell layer (curved arrowheads). The arrow indicates a leptomeningeal cell, also AQP1ext^+^. BS, brain stem; Cb, cerebellum; cp, cerebral peduncle; Ctx, cerebral cortex; Hip, hippocampus; Hyp, hypothalamus; OB, olfactory bulb; Pn, pontine nuclei. Scale bars: **a**, **b** 2 mm; **c**–**e** 100 μm; **f**–**j** 50 μm

**Fig. 5:**
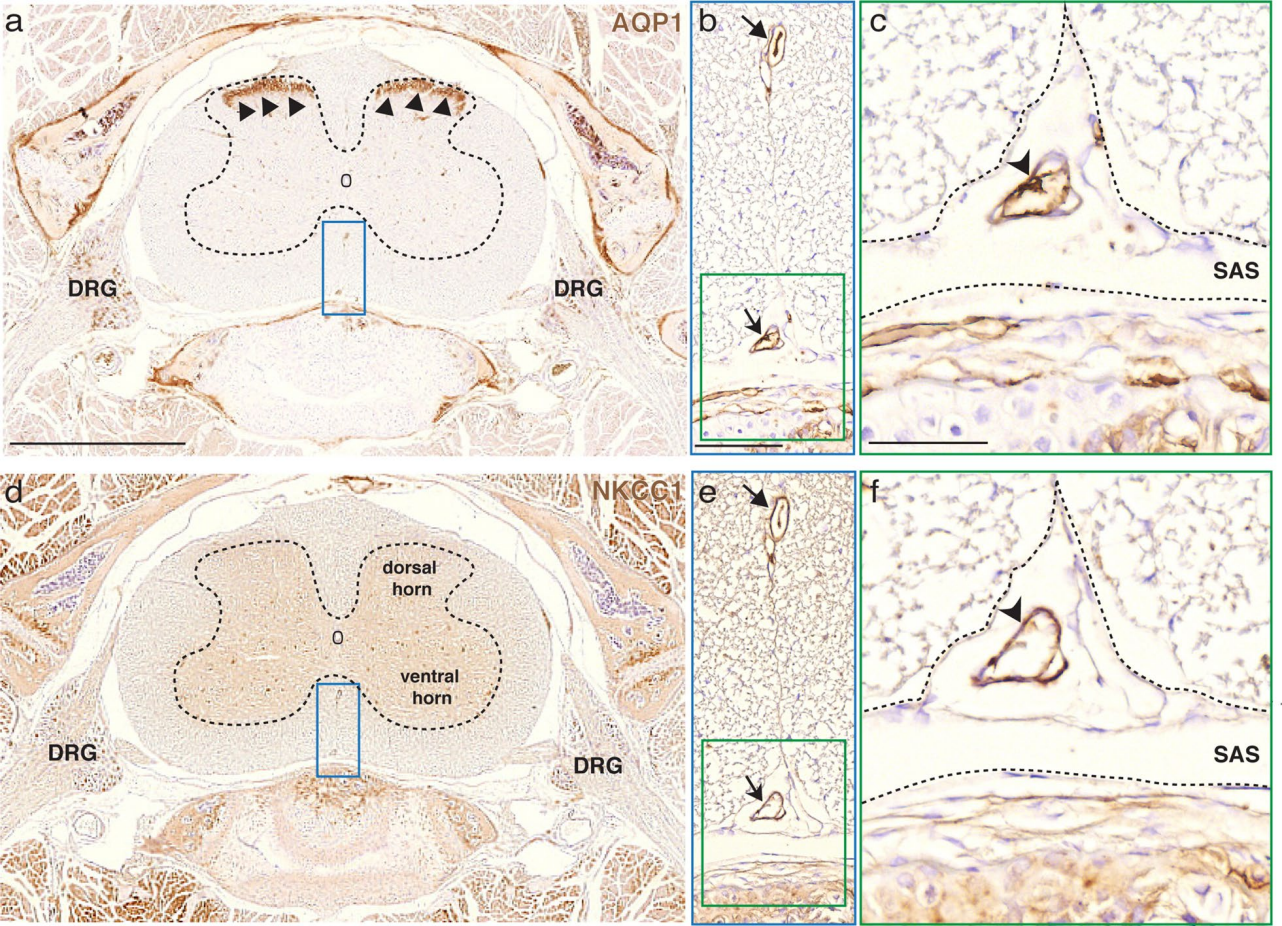
AQP1 and NKCC1 are present in leptomeningeal vascular endothelia of the spinal cord. Micrographs of paraffin sections obtained from the spinal cord of adult mice (P90) and immunolabeled for AQP1 and NKCC1 (brown). AQP1 immunoreactivity is predominantly located in C fibers in the dorsal horns of the spinal cord (**a**, arrowheads), whereas NKCC1 is observed throughout the spinal cord grey matter (**d**). **b**, **e** High magnification of the area delimited by the blue rectangle in **a** and **d**, respectively, show AQP1int^+^/NKCC1^+^ leptomeningeal vessels (arrows) in the spinal cord. **c**, **f** High magnification micrographs of the area delimited by the green squares in **b** and **e** show AQP1int^+^/NKCC1^+^ cells in the vascular endothelium, restricted to the subarachnoid space along the spinal cord (curved arrowheads). DRG, dorsal root ganglia; SAS, subarachnoid space. Scale bars: **a**, **d** 1 mm; **b**, **e** 100 μm; **c**, **f** 50 μm

An extra-choroidal source of CSF has been postulated to correspond to influx of fluid across the BBB [22]. Specifically, some CSF production has been suggested to occur at the capillary level, mediated by endothelial cells [4, 23]. However, there has been no investigation of this scenario using immunohistochemistry and light microscopy. Therefore, we asked if the endothelia of the leptomeningeal vasculature in the adult rodent CNS express AQP1 and NKCC1. The fine structure of capillaries and venules was accessed in paraffin sections obtained from adult mice (P60; n = 2 animals). We also assessed the distribution of AQP1 and NKCC1 in serial sections obtained from adult mouse brain (Fig. 4a, b). The vascular phenotype was determined in sections stained with haematoxylin (Fig. 4c). Higher magnification micrographs of the SAS, specifically at the *cisterna interpeduncularis* region, revealed AQP1int (Fig. 4d) and NKCC1 (Fig. 4e) immunoreactive cells in the smooth muscle cell layer of arterioles and in the endothelia of capillaries and venules. Confocal microscopy of brain sections from lectin-perfused mice showed that AQP1^+^ cells are restricted to the smooth muscle cell layer, and are absent in the leptomeningeal arterioles’ endothelia (Fig. 4f–j). In the leptomeningeal vasculature of the spinal cord, both AQP1 (Fig. 5a–c) and NKCC1 (Fig. 5d–f) were detected in the endothelial cells of the leptomeningeal capillaries.

## Discussion

We applied the uDISCO technique [43] to render the entire adult mouse head transparent, thus facilitating the brain-wide depiction of AQP1 immunoreactivity. This approach revealed the distribution of AQP1 in the vasculature of the intact brain, including the very fragile leptomeningeal vessels, which often become detached when the brain is removed from the skull (Fig. 1). AQP1 immunoreactive vascular profiles have been previously reported within the cerebral cortex of adult rats [63]. The diameters of such AQP1^+^ vessels corresponded mostly to arterioles, although some diameters were more consistent with capillaries. However, the AQP1 immunoreactivity was earlier described as occurring in discontinuous patches or zones along the vessels [63]. Our use of tissue clearing coupled to light-sheet microscopy proved greatly advantageous for establishing that AQP1 has continuous distribution along the leptomeningeal vasculature. Besides, tissue processing for immunohistochemistry, which entails the use of fixatives and pre-treatments such as dehydration and freezing, can modify the structure of epitopes and the availability of antibody-binding sites. We thus speculate that a proper appreciation of the AQP1 distribution in the leptomeningeal vasculature of adult rodents was hindered due to disruptive effects of histological processing.

The water channels formed by AQP1 are distributed predominantly in the apical but also in the basal membranes of epithelial cells in the choroid plexus, which positions them for an important role in CSF production. AQP1 allow bi-directional water movement in response to osmotic gradients, which are generated by ion pumps, transporters and co-transporters also present in the choroid plexus epithelia ([64]; reviewed in [13]). Nevertheless, transgenic mice lacking AQP1 have shown only a 20% decrease in CSF production [64] indicating that other proteins or mechanisms are involved in CSF production. Accordingly, the Na^+^/K^+^/2Cl^−^ cotransporter 1 (NKCC1) has been proposed as a main mediator of CSF production due to its capacity to couple water movement to ion translocation [31]. Thus, we sought in this study to determine if the distribution of NKCC1 correlates with that of AQP1 in the leptomeningeal vasculature.

While there is clear documentation of NKCC1 expression by endothelial cells cultured in vitro [59], the endothelia of arterioles and veins in the intact brain parenchyma is seemingly devoid of NKCC1 [62]. However, the brain capillary endothelia have been shown to contain NKCC1, mostly located at its luminal membrane, playing a role in stroke-induced edema [61] and in transendothelial ion uptake by the brain under ischemia [60]. Our observations indicate that cells positive for both AQP1 and NKCC1 localize to the smooth muscle layer of arterioles and veins (Figs. 2, 3), specifically residing within the SAS. Moreover, AQP1 and NKCC1 immunoreactive cells were also observed in the endothelia of veins, venules and capillaries distributed within the SAS (Fig. 4). Remarkably, AQP1^+^/NKCC1^+^ endothelial cells were also present in the leptomeningeal vasculature of the spinal cord (Fig. 5). We therefore suggest that the co-distribution of AQP1 and NKCC1 in the smooth muscle cell layer and in the capillary and venular endothelia in the adult rodent brain and spinal cord is a unique feature of leptomeningeal vessels.

Several studies in different mammalian species, including non-human primates, have challenged the classical view of the choroid plexus as the sole CSF source [4, 16–20]. Besides the ventricular system [1, 2], the SAS has been postulated as an important secondary source of CSF, producing as much as 40% of the total volume [18–20]. The results, based on measurements of CSF output, gave no insight into the molecular machinery supporting extra-choroidal CSF secretion. Our observations complement previous functional studies demonstrating that AQP1 and NKCC1, both proteins with acknowledged roles in CSF production by the choroid plexus (reviewed in [13]), are not expressed by parenchymal capillaries, but only by the leptomeningeal vasculature distributed in the SAS, including a subset of penetrating arterioles and veins. In the smooth muscle cell layer of the vasculature, AQP1 and NKCC1 co-distribution might participate in the maintenance of smooth muscle contractility [65], while also regulate transcellular transport of fluid [66]. At the capillary level, NKCC1^+^/AQP1^+^ endothelial cells of the SAS vasculature may contribute to the generation of osmotic gradients and facilitation of water movement. Present results partially corroborate the hypothesis that the vast capillary network of the CNS not only subserves oxygen and nutrient supply, but also produces ISF, which is ultimately incorporated into the total circulating CSF [4, 22–28]. Nevertheless, functional in vivo experiments with pharmacological or genetically-encoded alterations of AQP1 and NKCC1 expression and function, coupled to measurements of CSF production rate, are necessary to confirm that the leptomeningeal vasculature is an extra-choroidal source of CSF.

If proved correct, the existence of an extra-choroidal CSF source in the SAS vasculature may allow a comprehensive understanding of syndromes that relate to CSF production and circulation impairment, such as idiopathic intracranial hypertension (IIH) and idiopathic normal pressure hydrocephalus (iNPH), which are neurodegenerative diseases with non-determined causes [67, 68]. In patients with IIH syndrome, increase in the expression level of perivascular AQP4 correlates with the degree of astrogliosis resulting in increased fluid turnover by mechanisms that remain to be determined [69]. Alterations in fluid movement across the BCSFB along the SAS vasculature mediated by AQP1 and NKCC1, might relate to the changes in fluid turnover observed in this syndrome.

## Conclusions

**Fig. 6:**
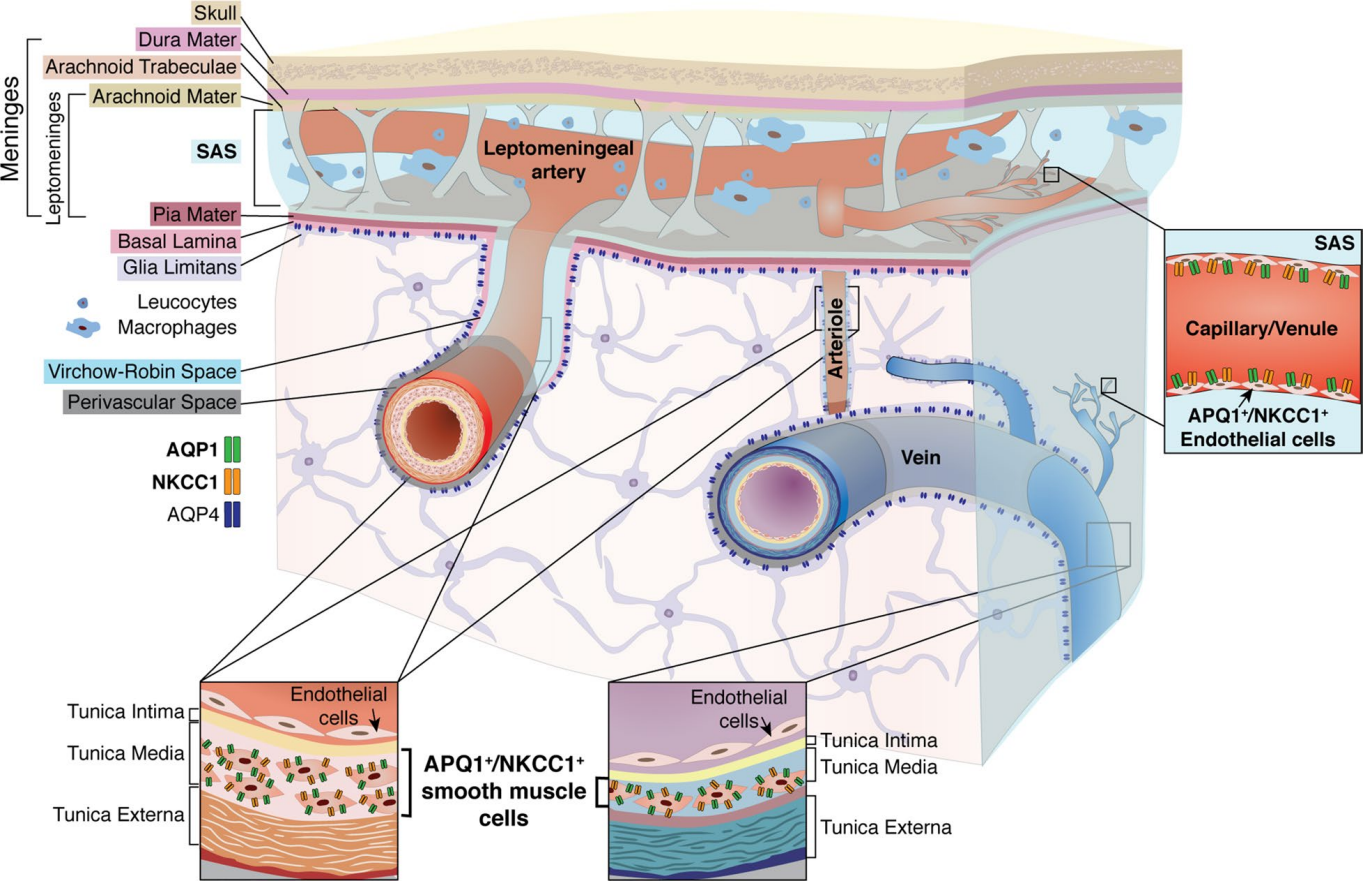
AQP1 and NKCC1 distribution in the CNS leptomeningeal vasculature. Scheme representing the mouse brain parenchyma, the skull and the meninges, which encompass the brain and also the spinal cord. The meninges are divided into the dura mater and the leptomeninges, corresponding to the arachnoid and pia mater. The brain and spinal parenchyma are separated from the meninges by the basal lamina and the glia limitans. The arachnoid mater forms the outer barrier of the CNS and underneath it lies the subarachnoid space (SAS), which is filled with CSF. Immune cells, namely macrophages and leucocytes, are sparsely present within the SAS, surveilling the healthy CNS. Additionally to its function as route for CSF and immune cells circulation, the SAS encloses the arterial blood supply to the CNS. Prior to entering the CNS parenchyma, leptomeningeal arteries branch and divide into arterioles. Within the parenchyma, penetrating arterioles and veins are tethered by astrocytes with highly polarized AQP4 distribution, a unique feature of the CNS vasculature. Schematic representation of cross sections of the leptomeningeal vasculature denotes AQP1 and NKCC1 expression by smooth muscle cells, which compose the tunica media of arterioles and veins. In contrast, endothelial cells within the tunica intima are devoid of both proteins. Notwithstanding, endothelial cells of capillaries and venules present both AQP1 and NKCC1

Herein we have described the distributions of AQP1 and NKCC1 in the leptomeningeal vasculature of the adult rodent brain and spinal cord. We confirmed our prediction of the presence of AQP1^+^ and NKCC1^+^ cells in the smooth muscle layer of the middle cerebral arteries (MCAs) and of arterioles and veins, as well as in the endothelia of capillaries and venules in the leptomeningeal vasculature throughout the CNS (Fig. 6). In contrast, the parenchymal vasculature is largely devoid of AQP1 and NKCC1, except for a subset of penetrating cortical arterioles. The distribution of AQP1 and NKCC1 in the endothelial cells of the capillaries present in the SAS posit them with the molecular setup that might contribute for extra-choroidal CSF production.

## Data Availability

The datasets used and/or analyzed during the current study are available from the corresponding author on reasonable request.

## Abbreviations

AQP1: Aquaporin 1
AQP4: Aquaporin 4
BBB: Blood-brain barrier
BCSFB: Blood-cerebrospinal fluid barrier
CSF: Cerebrospinal fluid
GAPDH: Glyceraldehyde 3-phosphate dehydrogenase
IIH: Idiopathic intracranial hypertension
iNPH: Idiopathic normal pressure hydrocephalus
ISF: Interstitial fluid
MCA: Middle cerebral arteries
NKCC1: Na^+^/K^+^/2Cl^−^ cotransporter 1
SAS: Subarachnoid space
*Slc12a2*: Solute carrier family 12 member 2
uDISCO: Ultimate three-Dimensional Imaging of Solvent-Cleared Organs

## References

[1] Dandy WE (1919). Experimental hydrocephalus. Ann Surg.

[2] Bering EA (1959). Cerebrospinal fluid production and its relationship to cerebral metabolism and cerebral blood flow. Am J Physiol.

[3] Milhorat TH (1975). The third circulation revisited. J Neurosurg.

[4] Segal MB, Pollay M (1977). The secretion of cerebrospinal fluid. Exp Eye Res.

[5] Veening JG, Barendregt HP (2010). The regulation of brain states by neuroactive substances distributed via the cerebrospinal fluid; a review. Cerebrospinal Fluid Res.

[6] Welch K (1963). Secretion of cerebrospinal fluid by choroid plexus of the rabbit. Am J Physiol Content.

[7] Ames A, Sakanoue M, Endo S (1964). Na, K, Ca, Mg, and Cl concentrations in choroid plexus fluid and cisternal fluid compared with plasma ultrafiltrate. J Neurophysiol.

[8] McComb JG (1983). Recent research into the nature of cerebrospinal fluid formation and absorption. Ann Surg.

[9] Davson H, Welch K, Segal MB (1987). The physiology and pathophysiology of the cerebrospinal fluid.

[10] Brown PD, Davies SL, Speake T, Millar ID (2004). Molecular mechanisms of cerebrospinal fluid production. Neuroscience.

[11] Praetorius J (2007). Water and solute secretion by the choroid plexus. Pflugers Arch Eur J Physiol.

[12] Brinker T, Stopa E, Morrison J, Klinge P (2014). A new look at cerebrospinal fluid circulation. Fluids Barriers CNS.

[13] Damkier HH, Brown PD, Praetorius J (2013). Cerebrospinal Fluid Secretion by the Choroid Plexus. Physiol Rev.

[14] Lun MP, Monuki ES, Lehtinen MK (2015). Development and functions of the choroid plexus–cerebrospinal fluid system. Nat Rev Neurosci.

[15] Spector R, Keep RF, Robert Snodgrass S, Smith QR, Johanson CE (2015). A balanced view of choroid plexus structure and function: focus on adult humans. Exp Neurol.

[16] Milhorat TH (1969). Choroid Plexus and Cerebrospinal Fluid Production. Science (80-).

[17] Milhorat TH, Hammock MK, Fenstermacher JD, Rall DP, Levin VA (1971). Cerebrospinal fluid production by the choroid plexus and brain. Science (80-).

[18] Bering EA, Sato O (1963). Hydrocephalus: changes in formation and absorption of cerebrospinal fluid within the cerebral ventricles. J Neurosurg.

[19] Sato O, Bering EA (1967). Extra-ventricular formation of cerebrospinal fluid. Brain Nerve.

[20] Sato O, Bering EAJ, Yagi M, Tsugane R, Hara M, Amano Y (1975). Bulk flow in the cerebrospinal fluid system of the dog. Acta Neurol Scand.

[21] Saunders NR, Dziegielewska KM, Møllgård K, Habgood MD (2018). Physiology and molecular biology of barrier mechanisms in the fetal and neonatal brain. J Physiol.

[22] Davson H, Segal MB (1970). The effects of some inhibitors and accelerators of sodium transport on the turnover of 22Na in the cerebrospinal fluid and the brain. J Physiol.

[23] Reese TS, Karnovsky MJ (1967). Fine structural localization of a blood-brain barrier to exogenous peroxidase. J Cell Biol.

[24] Bulat M, Klarica M (2011). Recent insights into a new hydrodynamics of the cerebrospinal fluid. Brain Res Rev.

[25] Orešković D, Klarica M (2010). The formation of cerebrospinal fluid: nearly a hundred years of interpretations and misinterpretations. Brain Res Rev.

[26] Orešković D, Klarica M (2011). Development of hydrocephalus and classical hypothesis of cerebrospinal fluid hydrodynamics: facts and illusions. Prog Neurobiol.

[27] Orešković D, Radoš M, Klarica M (2017). Role of choroid plexus in cerebrospinal fluid hydrodynamics. Neuroscience.

[28] Klarica M, Orešković D (2014). Enigma of cerebrospinal fluid dynamics. Croat Med J.

[29] Johanson CE, Duncan JA, Klinge PM, Brinker T, Stopa EG, Silverberg GD (2008). Multiplicity of cerebrospinal fluid functions: new challenges in health and disease. Cerebrospinal Fluid Res.

[30] Oldendorf WH (1977). The blood–brain barrier. Exp Eye Res.

[31] Steffensen AB, Oernbo EK, Stoica A, Gerkau NJ, Barbuskaite D, Tritsaris K (2018). Cotransporter-mediated water transport underlying cerebrospinal fluid formation. Nat Commun.

[32] Flagella M, Clarke LL, Miller ML, Erway LC, Giannella RA, Andringa A (1999). Mice lacking the basolateral Na–K–2Cl cotransporter have impaired epithelial chloride secretion and are profoundly deaf. J Biol Chem.

[33] Stagaard M, Balslev Y, Lundberg JJ, Mollgard K (1987). Microglia in the ependyma of the rat subcommissural organ following brain lesion with serotonin neurotoxin. J Neurocytol.

[34] Pérez E, Barrachina M, Rodríguez A, Torrejón-Escribano B, Boada M, Hernández I (2007). Aquaporin expression in the cerebral cortex is increased at early stages of Alzheimer disease. Brain Res.

[35] Cabral PD, Herrera M (2012). Membrane-associated aquaporin-1 facilitates osmotically driven water flux across the basolateral membrane of the thick ascending limb. Am J Physiol Ren Physiol.

[36] Kobayashi H, Yokoo H, Yanagita T, Satoh S, Kis B, Deli M (2006). Induction of aquaporin 1 by dexamethasone in lipid rafts in immortalized brain microvascular endothelial cells. Brain Res.

[37] Speake T, Freeman LJ, Brown PD (2003). Expression of aquaporin 1 and aquaporin 4 water channels in rat choroid plexus. Biochim Biophys Acta Biomembr.

[38] Owler BK, Pitham T, Wang D (2010). Aquaporins: relevance to cerebrospinal fluid physiology and therapeutic potential in hydrocephalus. Cerebrospinal Fluid Res.

[39] Skalli O, Ropraz P, Trzeciak A, Benzonana G, Gillessen D, Gabbiani G (1986). A monoclonal antibody against α-smooth muscle actin: a new probe for smooth muscle differentiation. J Cell Biol.

[40] Pusztaszeri MP, Seelentag W, Bosman FT (2006). Immunohistochemical expression of endothelial markers CD31, CD34, von Willebrand factor, and Fli-1 in normal human tissues. J Histochem Cytochem.

[41] Wu Y, Wu M, He G, Zhang X, Li W, Gao Y (2012). Glyceraldehyde-3-phosphate dehydrogenase: a universal internal control for Western blots in prokaryotic and eukaryotic cells. Anal Biochem.

[42] Nie X, Li C, Hu S, Xue F, Kang YJ, Zhang W (2017). An appropriate loading control for western blot analysis in animal models of myocardial ischemic infarction. Biochem Biophys Reports.

[43] Pan C, Cai R, Quacquarelli FP, Ghasemigharagoz A, Lourbopoulos A, Matryba P (2016). Shrinkage-mediated imaging of entire organs and organisms using uDISCO. Nat Methods.

[44] R Core Team (2017). R: A language and environment for statistical computing.

[45] Degasperi A, Birtwistle MR, Volinsky N, Rauch J, Kolch W, Kholodenko BN (2014). Evaluating strategies to normalise biological replicates of western blot data. PLoS ONE.

[46] Shields SD, Moore KD, Phelps PE, Basbaum AI (2010). Olfactory ensheathing glia express aquaporin 1. J Comb Neurol.

[47] Hasegawa H, Lian SC, Finkbeiner WE, Verkman AS (1994). Extrarenal tissue distribution of CHIP28 water channels by in situ hybridization and antibody staining. Am J Physiol.

[48] Praetorius J, Nielsen S (2006). Distribution of sodium transporters and aquaporin-1 in the human choroid plexus. Am J Physiol Cell Physiol.

[49] Johansson PA, Dziegielewska KM, Ek CJ, Habgood MD, Møllgård K, Potter A (2005). Aquaporin-1 in the choroid plexuses of developing mammalian brain. Cell Tissue Res.

[50] Yamamoto T, Sasaki S (1998). Aquaporins in the kidney: emerging new aspects. Kidney Int.

[51] Nielsen S, Smith BL, Christensen EI, Knepper MA, Agre P (1993). CHIP28 water channels are localized in constitutively water-permeable segments of the nephron. J Cell Biol.

[52] Nielsen S, Smith BL, Christensen EI, Agre P (1993). Distribution of the aquaporin CHIP in secretory and resorptive epithelia and capillary endothelia. Proc Natl Acad Sci USA.

[53] Verkman AS (2006). Aquaporins in endothelia. Kidney Int.

[54] Verkman AS, Anderson MO, Papadopoulos MC (2014). Aquaporins: important but elusive drug targets. Nat Rev Drug Discov.

[55] Butkus A, Alcorn D, Earnest L, Moritz K, Giles M, Wintour EM (1997). Expression of aquaporin-1 (AQP1) in the adult and developing sheep kidney. Biol Cell.

[56] Bedford JJ, Leader JP, Walker RJ (2003). Aquaporin expression in normal human kidney and in renal disease. J Am Soc Nephrol.

[57] Bairamian D, Johanson CE, Parmelee JT, Epstein MH (1991). Potassium cotransport with sodium and chloride in the choroid plexus. J Neurochem.

[58] Keep RF, Xiang J, Betz AL (1994). Potassium cotransport at the rat choroid plexus. Am J Physiol Cell Physiol.

[59] Sun D, Lytle C, O’Donnell ME (1995). Astroglial cell-induced expression of Na-K-Cl cotransporter in brain microvascular endothelial cells. Am J Physiol.

[60] Brillault J, Lam TI, Rutkowsky JM, Foroutan S, O’Donnell ME (2008). Hypoxia effects on cell volume and ion uptake of cerebral microvascular endothelial cells. Am J Physiol Cell Physiol.

[61] O’Donnell ME, Tran L, Lam TI, Liu XB, Anderson SE (2004). Bumetanide inhibition of the blood-brain barrier Na–K–Cl cotransporter reduces edema formation in the rat middle cerebral artery occlusion model of stroke. J Cereb Blood Flow Metab.

[62] Plotkin MD, Kaplan MR, Peterson LN, Gullans SR, Hebert SC, Delpire E (1997). Expression of the Na(+)-K(+)-2Cl^−^ cotransporter BSC2 in the nervous system. Am J Physiol.

[63] Wilson AJ, Carati CJ, Gannon BJ, Haberberger R, Chataway TK (2010). Aquaporin-1 in blood vessels of rat circumventricular organs. Cell Tissue Res.

[64] Oshio K, Watanabe H, Song Y, Verkman AS, Manley GT (2005). Reduced cerebrospinal fluid production and intracranial pressure in mice lacking choroid plexus water channel aquaporin-1. FASEB J.

[65] Garg P, Martin CF, Elms SC, Gordon FJ, Wall SM, Garland CJ (2007). Effect of the Na-K-2Cl cotransporter NKCC1 on systemic blood pressure and smooth muscle tone. Am J Physiol Circ Physiol.

[66] Shanahan CM, Connolly DL, Tyson KL, Cary NRB, Osbourn JK, Agre P (1999). Aquaporin-1 is expressed by vascular smooth muscle cells and mediates rapid water transport across vascular cell membranes. J Vasc Res.

[67] Adams RD, Fisher CM, Hakim S, Ojemann RG, Sweet WH (1965). Symptomatic occult hydrocephalus with normal cerebrospinal-fluid pressure. N Engl J Med.

[68] Ball AK, Clarke CE (2006). Idiopathic intracranial hypertension. Lancet Neurol.

[69] Eidsvaag VA, Hansson H-A, Heuser K, Nagelhus EA, Eide PK (2018). Cerebral microvascular abnormalities in patients with idiopathic intracranial hypertension. Brain Res.

